# Monitoring air quality index with EWMA and individual charts using XGBoost and SVR residuals

**DOI:** 10.1016/j.mex.2024.103107

**Published:** 2024-12-12

**Authors:** Zulfani Alfasanah, M. Zaim Husnun Niam, Sri Wardiani, Muhammad Ahsan, Muhammad Hisyam Lee

**Affiliations:** a Department of Statistics, Institut Teknologi Sepuluh Nopember, Indonesia; b Department of Mathematical Sciences, Universiti Teknologi Malaysia, Johor Bahru, Malaysia

**Keywords:** PM2.5, Air pollution, XGBoost, Support vector regression, EWMA chart, Individual chart, Jakarta, EWMA, Individuals Chart, XGBoost, SVR

## Abstract

PM2.5 air pollution poses significant health risks, particularly in urban areas such as Jakarta, where concentrations frequently surpass acceptable levels due to rapid urbanization. This study addresses autocorrelation in air quality data and evaluates the monitoring performance of XGBoost and Support Vector Regression (SVR) models using Individual and Exponentially Weighted Moving Average (EWMA) Charts. PM2.5 levels were obtained from Jakarta's Air Quality Index. The findings reveal that the SVR model effectively manages autocorrelation, while the combination of XGBoost and the EWMA chart yielded superior monitoring performance. Specifically, this approach detected only one out-of-control (OOC) point in Phase II and none in Phase I, with identified shifts ranging from moderate to large. Overall, the XGBoost and EWMA chart integration offers a robust solution for precise air quality monitoring and minimizes false alarms. The identification of OOC points provides actionable insights by highlighting significant deviations in air quality data that may require immediate intervention.

Key points:

• SVR and XGBoost model regression was introduced to enhance forecasting accuracy.
• EWMA chart based on XGBoost residuals has better monitoring results.

## Graphical abstract

**Figure:**
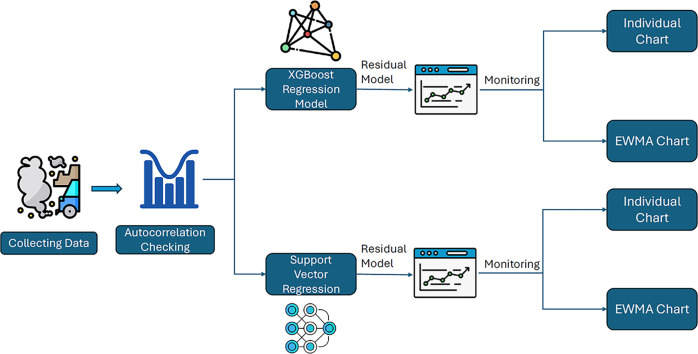

Specifications table

This table provides general information on your method.

**Table:** 

Subject area:	Mathematics and Statistics
More specific subject area:	*Statistical Process Control, Autocorrelation Data*
Name of your method:	EWMA, Individuals Chart, XGBoost, SVR
Name and reference of original method:	*NA]*
Resource availability:	*R Studio*

## Background

Air pollution levels have become a global concern due to their harmful effects on human health and the environment. Among the various types of pollutants, PM2.5 is one of the most dangerous [1]. PM2.5 consist of particles with a diameter of <2.5 µm [2]. Accurate prediction of spatial variability in PM2.5 concentrations using high-performance models is essential for the prevention and management of air pollution [3]. Short-term and long-term exposure to PM2.5 is associated with increased risks of heart attacks, asthma, and premature death [4]. A significant `portion of the population, including children, individuals with heart or lung conditions, and minority groups, are particularly vulnerable to the health effects of PM2.5 [5]. These studies indicate that PM2.5 air pollution is not only linked to health issues, but also contributes to climate change. Fine particulate matter (PM2.5), which consists of particles with an aerodynamic diameter of 2.5 mm or less, has consistently been one of China's main pollutants due to decades of rapid economic growth. Since 2013, the Chinese government has included PM2.5 in the ground automatic monitoring network, enabling accurate hourly measurements of PM2.5 concentrations. PM2.5 has garnered significant attention from both governmental bodies and researchers [6]. This urgency is mirrored in Indonesia, particularly Jakarta, where poor air quality exacerbated by high PM2.5 levels requires improved air pollution mitigation strategies and predictive models.

In Indonesia, specifically Jakarta, poor air quality is a significant issue, exacerbated by recent data indicating concerning PM2.5 levels, particularly during periods of low precipitation [7]. This situation necessitates improved strategies for air pollution mitigation and the development of better predictive models to effectively manage air quality [8]. Jakarta's rapid urbanization has resulted in recurrent violations of PM2.5 air quality standards, presenting substantial health hazards. This alarming situation highlights that air pollution not only poses significant public health challenges but also adversely impacts the quality of life for its residents. Jakarta has experienced rapid growth economic growth and urbanization, leading to increased air pollution. The Air Quality Index has conducted extensive research on air pollution in Jakarta, providing daily data to monitor the high levels of particulate matter. Recent reports indicate that children and individuals with pre-existing health conditions, such as cardiovascular and pulmonary diseases, are particularly susceptible to the harmful effects of PM2.5. Therefore, it is crucial to strengthen air pollution mitigation efforts in Jakarta, considering the elevated mortality rates associated with respiratory issues, especially among vulnerable populations [9]. The government has made numerous efforts to monitor and control air pollution levels, such as implementing the odd-oven license plate system. However, conventional monitoring methods often fail to detect small and gradual changes in pollutants data effectively. The pressing need for effective monitoring and management is further emphasized by findings in prior studies assessing climate models and their implications for environmental forecasting, especially in similar tropical climates. Moreover, recent works have demonstrated the effectiveness of artificial intelligence-based techniques in various environmental forecasting contexts, including rainfall forecasting, suggesting that similar methodologies could enhance air quality predictions [10].

Acknowledging the limitations of conventional monitoring techniques in capturing the nuances of air quality fluctuations,this research can focuses on developing a hybrid model incorporating Extreme Gradient Boosting (XGBoost), GARCH models, and a Multilayer Perceptron (MLP) neural network to predict PM2.5 concentration and volatility, thereby addressing the inherent autocorrelation challenges within air quality datasets [11]. Substantial evidence links both short-term and long-term exposure to PM2.5 with heightened morbidity and mortality rates, particularly in reference to respiratory diseases. In this study, the limitations of available data and the computational demands of LSTM indicated that XGBoost and SVR were more appropriate choices for accurately capturing PM2.5 fluctuations. Therefore, XGBoost and SVR were identified as more suitable options for accurately capturing the fluctuations in PM2.5 levels. The robustness of our study is highlighted by its holistic approach, as the combination of XGBoost the effective identification of both spatial and temporal patterns in the data, handling outliers,an aspect often missed by conventional techniques and building on recent advancements in environmental forecasting, including the application of artificial intellegence in predicting air quality to contribute the development of more machine learning predictive model [12,13]. To evaluate model performance, a comparative analysis employing Exponentially Weighted Moving Average (EWMA), Individual Control Charts, and a benchmark established through Extreme Gradient Boosting (XGBoost) and Support Vector Regression (SVR) will be conducted.

In the analysis of ecological and environmental data, observations frequently exhibit temporal dependence, manifesting as correlations or autocorrelations. This inherent structure violates the assumptions of independence and normality underlying traditional Shewhart control charts, rendering them inapplicable. To address this challenge, researchers have employed control chart methodologies such as the Exponentially Weighted Moving Average (EWMA) chart and the control chart individuals method for real-time detection and monitoring of data variations. The decision to utilize Exponentially Weighted Moving Average (EWMA) and Individual Control Charts is driven by their strength in detecting subtle data pattern changes, especially in autocorrelated datasets. EWMA effectively identifies small shifts due to its weighting scheme, proving its robustness in environmental monitoring. It is well-suited for real-time PM2.5 monitoring, demonstrating high sensitivity to minor variations [2]. The advantages of EWMA for quality control further emphasize its necessity in air quality assessments and support its inclusion in predictive models for pollution monitoring [14]. Overall, EWMA plays a critical role in maintaining accuracy and reliability in environmental data series [15,16]. The decision to utilize EWMA and Individual Control Charts was based on their strengths in detecting subtle changes in data patterns, particularly when dealing with autocorrelated datasets. The EWMA chart is particularly effective in identifying small shifts due to its weighting scheme, while Individual Control Charts are easily interpretable in monitoring single observations. Although alternative methods such as Cumulative Sum (CUSUM) charts may offer robust options for monitoring changes in process means, the focus here is on the ability of EWMA to continuously monitor PM2.5 levels with sensitivity to small variations [17]. Notably, Supharakonsakun et al. evaluated the performance of a modified EWMA control chart for monitoring autocorrelated PM2.5 and CO data. Their findings demonstrated the effectiveness of this approach in identifying subtle changes within air pollution datasets [2]. EWMA control charts are recommended for monitoring time series data exhibiting non-normality or autocorrelation, as substantiated by Average Run Length (ARL) analysis. To forecast PM2.5 concentrations, models such as XGBoost and Support Vector Regression (SVR) were utilized, leveraging historical data alongside meteorological variables. Previous studies have validated the efficacy of XGBoost and SVR in PM2.5 concentration forecasting. In alignment with these findings, demonstrated the exceptional performance of XGBoost in predicting PM2.5 concentrations using satellite and meteorological data, achieving an R² value of 0.81 [18]. The study demonstrates that the proposed QPSO-SVR model surpasses alternative models in terms of predictive accuracy and computational efficiency. Moreover, it exhibits enhanced robustness to meteorological influences when forecasting PM2.5 and NO2 concentrations [19].

This investigation is projected to expand the current understanding of PM2.5 air pollution, thereby providing a foundation for implementing strategies to ameliorate Jakarta's air quality. Building upon the established research framework, the primary objectives of this study encompass the creation of a model robust to autocorrelation within the dataset, a comprehensive analysis of residuals generated by the superior models, XGBoost and Support Vector Regression (SVR), and a comparative evaluation of the monitoring capabilities exhibited by Individual and Exponentially Weighted Moving Average (EWMA) Charts.

## Method details

### Data collection

Air quality were sourced from the AQICN platform for the period spanning January 1, 2023 to May 31, 2024. The dataset encompassed Particulate Matter 2.5 (PM2.5) concentrations, a primary indicator of air quality. To facilitate temporal analysis, the data were partitioned into two phases: Phase I (training phase), comprising January 1, 2023, to December 31, 2023, and Phase II, encompassing January 1, 2024 to May 31, 2024. The training data captured all seasons in Indonesia, a tropical country with a dry and rainy season.

### Data preprocessing

To address autocorrelation in the data, we first examined the Autocorrelation Function (ACF) plot of the PM 2.5 data to identify whether there was a relationship between data values at close time intervals. Next, to address autocorrelation, modeling was performed using XGBoost and SVR (Support Vector Regression), incorporating significant lags. These significant lags were determined by examining the results of the Partial Autocorrelation Function (PACF) plot, which helps identify the number of lags that should be included in the model to capture relevant time dependencies. By using the significant lags from the PACF, XGBoost and SVR models were built to predict the air quality index more accurately, without being affected by unwanted autocorrelation.

### Model development

#### Support vector regression

Support Vector Regression (SVR) is a regression algorithm derived from the Support Vector Machine (SVM) framework introduced by Vapnik in 2000. Fundamentally, SVR employs a non-linear mapping to project data from a lower-dimensional input space into a higher-dimensional feature space [20]. Within this augmented feature space, a regression model is constructed. Support Vector Regression (SVR) employs Vapnik's epsilon-insensitive loss function and structural risk minimization to minimize the expected approximation error from a finite sample. SVR maps input data nonlinearly into a high-dimensional feature space, enabling linear model representation of complex relationships [21]. Support Vector Regression (SVR) aims to identify a function f(x) that optimally interpolates training data points x, maximizing the margin between the predicted values and the observed target values y. The SVR framework models both input patterns x and the target function f within the context of Support Vector Machines. Fundamentally, SVR constructs a regression model characterized by an epsilon-insensitive loss function, thereby minimizing prediction errors for unseen data [22]. The regression function in Support Vector Regression can be mathematically expressed in Eq. (1).

(1) $$f\left({\overset{⇀}{x}}_{t}\right)=\sum_{t=1}^{T}\left(\beta_{t}-\beta_{t}^{*}\right)K\left({\overset{⇀}{x}}_{t},{\overset{⇀}{x}}_{j}\right)+b$$

${\overset{⇀}{x}}_{t}=\left\{x_{1,t},x_{2,t},x_{3,t},...,x_{i,t}\right\}\in R^{i}$ is a vector in the input space with i representing the number of input variables, $\beta_{t}$ and $\beta_{t}^{*}$ are optimization parameters obtained from the dual solution in SVR. $K\left({\overset{⇀}{x}}_{t},{\overset{⇀}{x}}_{j}\right)=\varphi \left({\overset{⇀}{x}}_{t}\right)\varphi \left({\overset{⇀}{x}}_{j}\right)$ is the kernel function used to map the data ${\overset{⇀}{x}}_{t}$ from the input space to a higher-dimensional feature space of a function φ that becomes $\varphi :{\overset{⇀}{x}}_{t}\to \varphi \left({\overset{⇀}{x}}_{j}\right)$, and b is the bias. A variety of kernel functions are commonly employed in SVR including the Linear, Radial Basis Function (RBF), Polynomial, and Sigmoid kernels [23]. Given the inherently non-linear nature of many time series, dynamic modeling approaches such as polynomial and Radial Basis Function (RBF) methods have gained prominence. Nevertheless, linear models remain valuable for initial exploration of data linearity and comparative analysis. This study will employ linear, RBF, and polynomial kernel methods to investigate the underlying structure of the time series data according to Eqs. (2)–(4).

(2) $$K\left({\overset{⇀}{x}}_{t},{\overset{⇀}{x}}_{j}\right)={\overset{⇀}{x}}_{t}^{T}{\overset{⇀}{x}}_{j}$$

(3) $$K\left({\overset{⇀}{x}}_{t},{\overset{⇀}{x}}_{j}\right)=\exp\left(-\frac{1}{2\sigma^{2}}∥{\overset{⇀}{x}}_{t}-{\overset{⇀}{x}}_{j}∥{;}^{2}\right)$$

(4) $$K\left({\overset{⇀}{x}}_{t},{\overset{⇀}{x}}_{j}\right)={\left({\overset{⇀}{x}}_{t}{\overset{⇀}{x}}_{j}^{T}+r\right)}^{d}$$

${\overset{⇀}{x}}_{t}=\left\{x_{1,t},x_{2,t},x_{3,t},...,x_{i,t}\right\}\in R^{i}$ is the first vector in the input space with i representing the number of input variables, ${\overset{⇀}{x}}_{j}=\left\{x_{1,j},x_{2,j},x_{3,j},...,x_{k,j}\right\}\in R^{k}$ is the second vector in the input space with k representing the number of input variables, $K\left({\overset{⇀}{x}}_{t},{\overset{⇀}{x}}_{j}\right)=\varphi \left({\overset{⇀}{x}}_{t}\right)\varphi \left({\overset{⇀}{x}}_{j}\right)$ is the kernel function used to map the data ${\overset{⇀}{x}}_{t}$ from the input space to a higher-dimensional feature space of a function φ that becomes $\varphi :{\overset{⇀}{x}}_{t}\to \varphi \left({\overset{⇀}{x}}_{j}\right)$, σ is a scale parameter, r is a free constant added to control the shift in feature space, and d is the polynomial degree. To Select the optimal parameter for SVR forecasting, hyperparameter tuning can be employed to train the data [24]. Identifying the best parameters through hyperparameter optimization may utilize grid search combined with cross-validation techniques. For time series data, cross-validation is conducted by splitting a portion of Phase I data into validation set while preserving the chronological order of the data. The result of the grid search, which produces the smallest error value, will be used as the hyperparameter input for the SVR model to predict the data [25].

#### Xtreme gradient boosting regression

Chen and Guestrin introduced the Extreme Gradient Boosting (XGBoost) algorithm in 2016. Widely regarded as a state-of-the-art ensemble learning method based on decision trees, XGBoost has garnered significant attention within the data science community. This algorithm employs a gradient boosting framework, sequentially constructing models to predict the residuals of preceding models. By optimizing the model parameters through gradient descent, XGBoost effectively minimizes prediction errors at each iteration [26]. Gradient boosting iteratively minimizes a loss function, commencing with an initial function F_0_(x). At each iteration, the algorithm seeks to reduce the value of the loss function through successive approximations.

(5) $$\left\{\gamma_{j},h_{j}\right\}=\arg\;\min\;\sum_{j=1}^{J}L(y_{i},f^{\left(j-1\right)}\left(x_{i}\right)+\gamma_{j}h_{j}\left(x_{i}\right))$$

$\gamma_{j}$ is a scalar that controls the magnitude of the contribution from the additional model $h_{j}$ at iteration j, $y_{i}$ is the actual output of data point i, and $L(y_{i},f^{\left(j-1\right)}\left(x_{i}\right)+\gamma_{j}h_{j}\left(x_{i}\right))$ is the loss function that measures how well the prediction from the current model aligns with the actual target $y_{i}$.

When observational data are not independent, conventional control charts may not function correctly. To address autocorrelated data, the XGBoost regression model can be used. Let $y_{1},y_{2},...,y_{j}$ be the output observation vectors, representing autocorrelated multivariate data, where $y_{m}={\left(y_{1m},y_{2m},...,y_{nm}\right)}^{T}$ with m=1,2,...,jiIndicates the cardinality of the XGBoost ensemble. Each constituent output vector is hypothesized to exhibit significant Partial Autocorrelation (PACF) coefficients for lags up to a specified order — $t_{1},t_{2},...,t_{j}$. Thus, the input for the XGBoost model is defined accordingly.

(6) $$x=\left(y_{1,(i-1)},...,y_{1,(i-t_{1})},...,y_{j,(i-1)},...,y_{j,(i-t_{j})}\right)$$

x is the input vector for the XGBoost model consisting of observed values taken at several previous lags or time intervals and $\left(y_{1,(i-1)},...,y_{1,(i-t_{1})},...,y_{j,(i-1)},...,y_{j,(i-t_{j})}\right)$ is components of the input vector x containing the output observation vectors at various time lags. XGBoost is a prominent supervised learning algorithm distinguished by its ensemble structure, which incorporates an objective function and base learners to optimize predictive performance [27]. The objective function in XGBoost is composed of a loss function quantifying the discrepancy between observed and predicted outcomes, and a regularization term penalizing model complexity. To enhance predictive accuracy, XGBoost employs ensemble learning, aggregating predictions from multiple base models. This approach mitigates the impact of individual model errors. A regressor constructs a statistical model based on input features to estimate corresponding output values.

In this study, the models were built using XGBoost with a maximum tree depth of 3 and 50 boosting rounds (nrounds). These hyperparameters were carefully selected to balance model complexity and generalization. The relatively shallow depth of 3 was chosen to prevent overfitting by restricting the depth of the decision trees, allowing the model to capture key patterns without being overly influenced by data noise. The 50 boosting iterations were set to ensure enough rounds for the model to reach a stable solution while minimizing computational cost and reducing the risk of overfitting.

#### EWMA control chart

The Exponentially Weighted Moving Average (EWMA) control chart is a statistical process control technique particularly effective in detecting small process shifts relative to the Shewhart control chart. Applicable to both attribute and variable data under assumptions of normality and subgroup homogeneity, the EWMA incorporates information from historical data into the calculation of each plotted point, unlike the Shewhart chart which relies solely on the current sample. This method can be employed for individual observations or subgroups with sample sizes greater than one. The EWMA is mathematically defined as follows [28].

(7) $$Z_{k}=\lambda x_{k}+\left(1-\lambda \right)Z_{k-1}$$

Where Z_k_ is the value of the production result, λ is the weighting parameter, and x_k_ is the k-th observed value, with *k* = 1,2,3,…,n representing time or observation subgroups. The weighting parameter has a constant value of 0 < λ ≤ 1, and Z_0_ is the initial or expected value of the production result, thus Z_0_ = µ_0_. Sometimes, Z_0_ can be derived from the average of the observed values, $Z_{0}=\bar{X}$. The center line and control limits for the EWMA control chart are as follows, with L representing the width of the control limits and σ is the standard deviation of the process in a stable condition.

(8) $$UCL=\mu_{0}+L\sigma \sqrt{\frac{\lambda }{\left(2-\lambda \right)}\left[1-{\left(1-\lambda \right)}^{2k}\right]}$$

(9) $$CL=\mu_{0}$$

(10) $$LCL=\mu_{0}-L\sigma \sqrt{\frac{\lambda }{\left(2-\lambda \right)}\left[1-{\left(1-\lambda \right)}^{2k}\right]}$$

As k approaches infinity, the control limits will stabilize and become as shown below.

(11) $$UCL=\mu_{0}+L\sigma \sqrt{\frac{\lambda }{\left(2-\lambda \right)}}$$

(12) $$LCL=\mu_{0}-L\sigma \sqrt{\frac{\lambda }{\left(2-\lambda \right)}}$$

The Exponentially Weighted Moving Average (EWMA) chart's design is characterized by two primary parameters: the sigma multiplier for control limits (L) and the weighting factor (λ). Optimal selection of L and λ is crucial for attaining the desired Average Run Length (ARL). The selection of these two parameters is important because EWMA is capable of detecting small shifts. Therefore, this method is suitable for cases with frequent changes, such as air quality monitoring. Empirical evidence suggests that a weighting factor within the interval 0.05 ≤ λ ≤ 0.25 generally yields satisfactory performance. The EWMA is a prevalent technique in time series analysis and prediction [29]. The EWMA's computation, which incorporates a weighted average of historical and current data points, renders it relatively insensitive to departures from normality. Consequently, it is a robust control charting technique for monitoring individual observations.

#### Individual control chart

In numerous instances, process monitoring is conducted using sample sizes (n) of one, wherein each individual unit constitutes a data point. Time series data, for example, often adheres to this paradigm. Given the application of automated inspection and measurement technologies, each observation is independently analyzed, rendering the formation of rational subgroups impractical. Under such circumstances, the Shewhart individuals control chart emerges as a suitable statistical process control tool. To estimate process variability within this context, the moving range between successive observations is commonly employed. Consider $x_{k}$, k=1,2,..., as individual observations from a process concerning a specific quality characteristic of interest. The moving range is defined as follows [28].

(13) $$MR_{k}=\left|x_{k}-x_{k-1}\right|$$

The parameters for the control chart designed for individual measurements are as follows.

(14) $$UCL=\bar{x}+3\frac{\bar{MR}}{d_{2}}$$

(15) $$CL=\bar{x}$$

(16) $$LCL=\bar{x}-3\frac{\bar{MR}}{d_{2}}$$

where $\bar{x}$ is the average of all individual values observed in the data, $\bar{MR}$ is the average of the moving range calculated from an initial set of data, and $d_{2}$ are control chart constants for *n* = 2, with values commonly found in most quality control textbooks. The interpretation of the individual control chart closely resembles that of the conventional $\bar{x}$ control chart. A deviation in the process mean from its stable state manifests as one or more data points exceeding the control limits on an individual control chart. The Shewhart individuals control chart is a robust and straightforward method for assessing process stability [30].

**Fig. 1:**
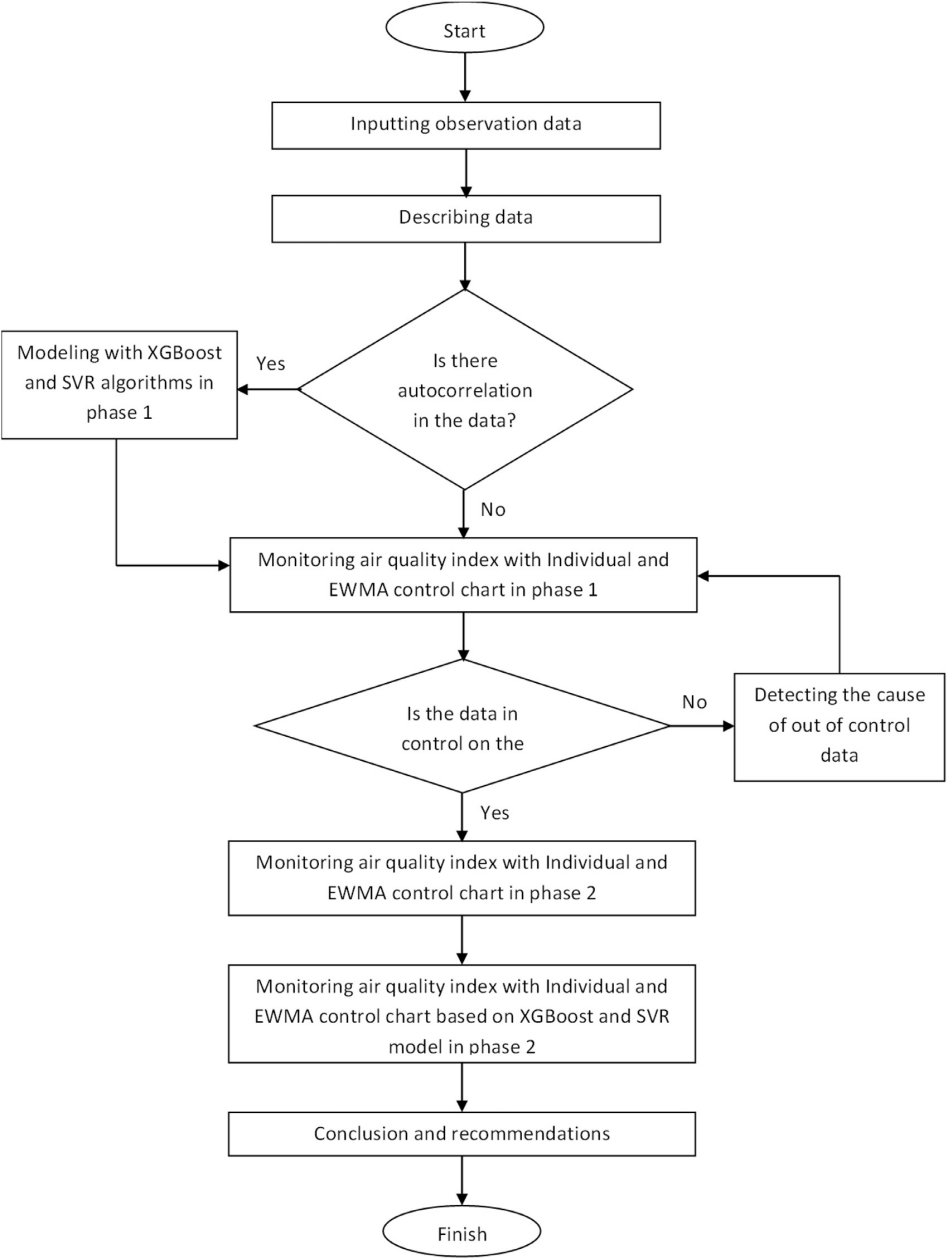
Monitoring process Flowchart.

The research methodology follows a sequential process, outlined through the stages depicted in the accompanying flowchart in Fig. 1.

## Method validation

### Implementation platform details

The system's implementation is based on R statistical software, version 4.1.2. The analysis pipeline incorporates the caret, xgboost, e1071, kernlab, qcc, and ggplot2 packages. Key packages were selected to optimize the analysis: caret for model prepocessing and validation, xgboost for efficient XGBoost time series modeling, e1071 and kernlab for advanced SVR time series modeling, qcc for EWMA and Individual control chart analysis, and ggplot2 for effective data visualization. This comprehensive toolkit enabled a detailed and replicable analysis process.

### Model evaluation

**Fig. 2:**
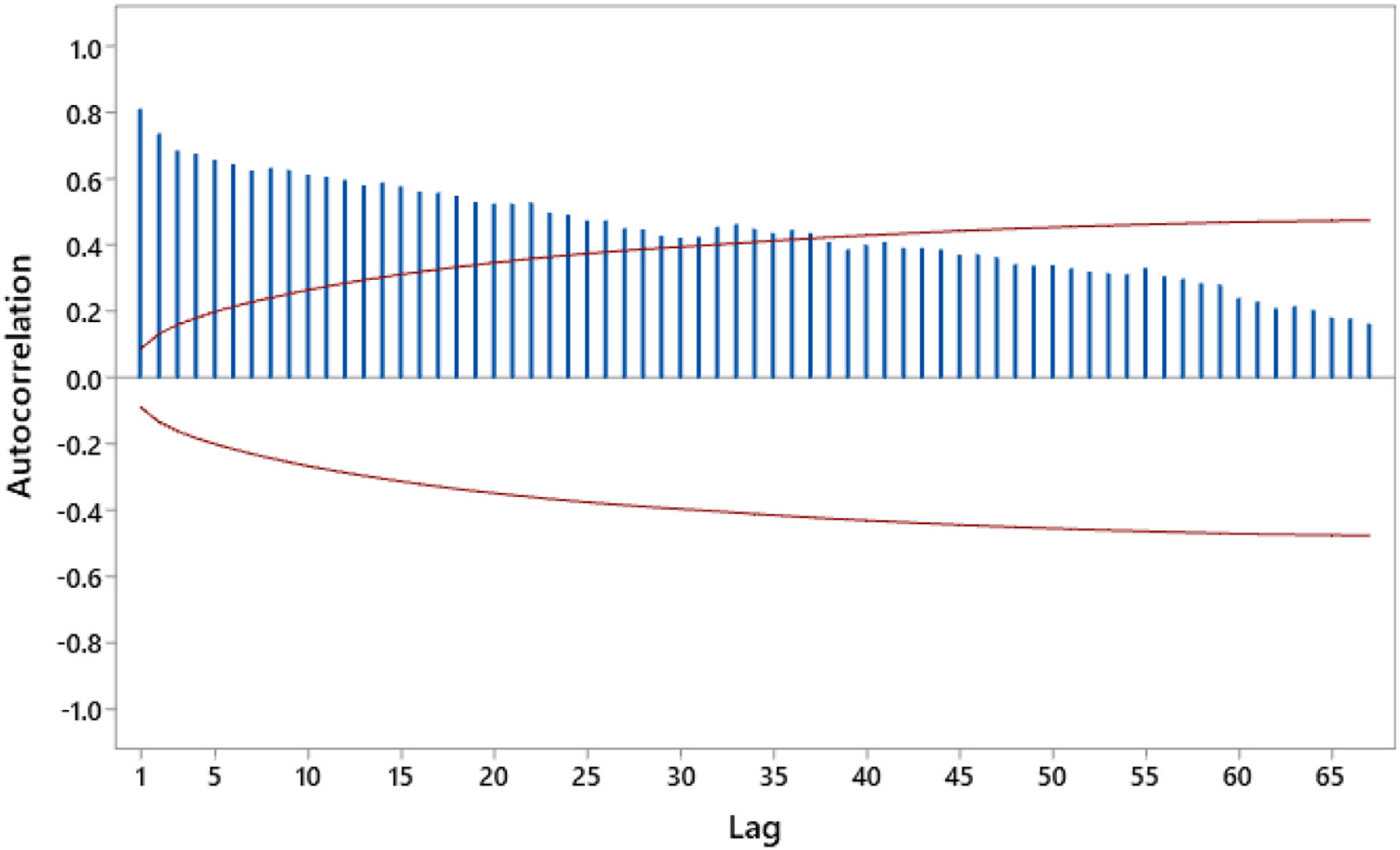
ACF Plot of air quality index data based on PM 2.5 in Jakarta.

Fig. 2 shows the ACF plot of the obtained data.

**Fig. 3:**
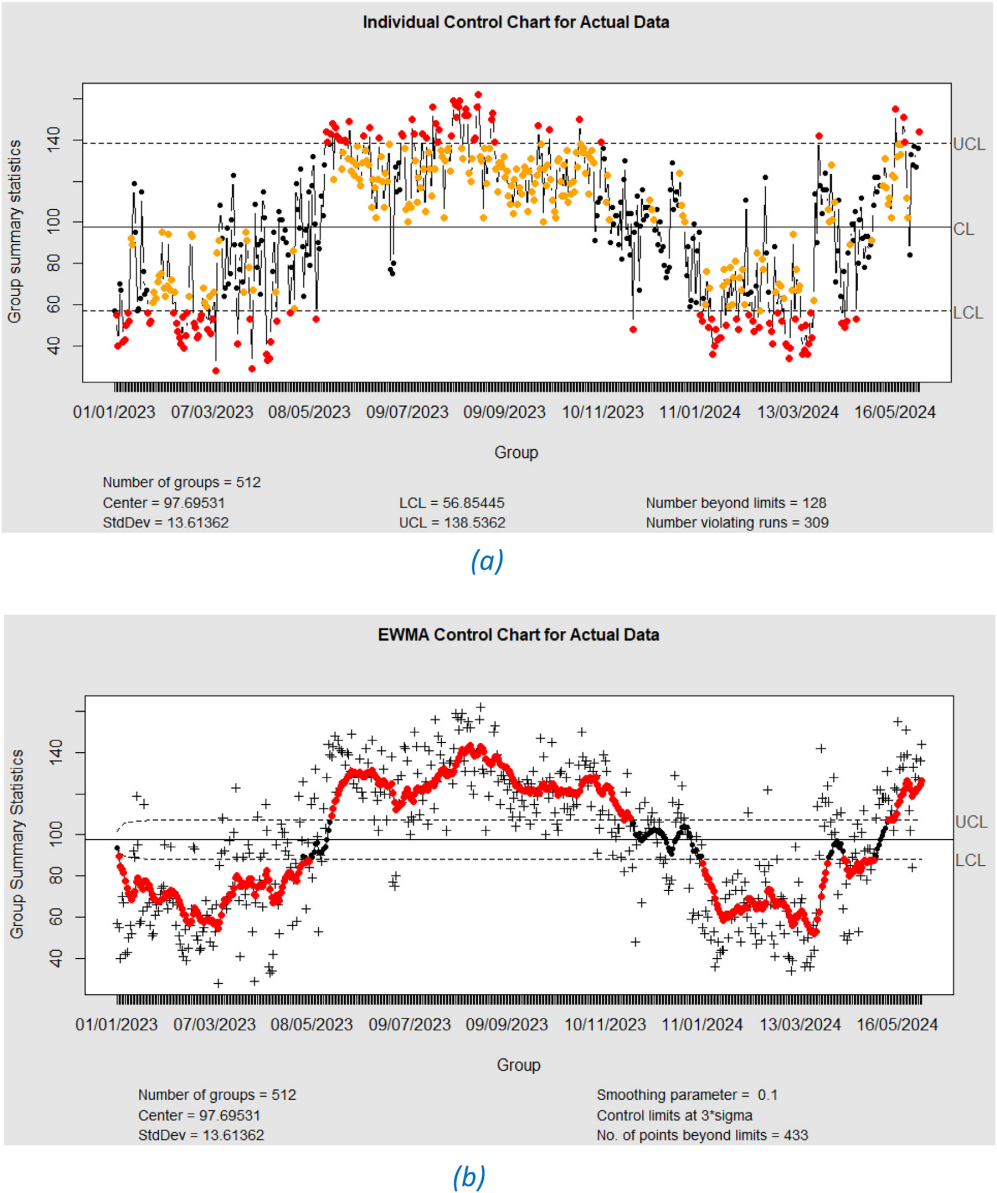
Control Chart for Actual Data. (a) Individual Control Chart, (b) EWMA Control Chart.

Fig. 2 illustrates pronounced autocorrelation in Jakarta's PM2.5-based Air Quality Index (AQI) data for lags 1 to 37. This temporal dependency is inherent to the daily time series nature of the AQI observations. To account for the autocorrelation structure, XGBoost and Support Vector Regression (SVR) models were employed for the analysis of the raw data. The presence of autocorrelation is further corroborated by the EWMA and individual control charts depicted in Fig. 3.

An analysis of PM2.5 air quality data in Jakarta, visualized through control charts in Fig. 3, reveals pronounced evidence of autocorrelation. Specifically, Figs. 3(a) and 2(b) exhibit 128 and 433 data points, respectively, exceeding the upper and lower control limits (UCL and LCL), indicating a statistically non-conforming process. The presence of extended patterns of data points above or below the center line further corroborates the existence of autocorrelation, a common characteristic of time series data where observations are sequentially dependent. Such autocorrelation can lead to erroneous interpretations of process behavior through control charts, manifesting as spurious alarms or missed process shifts. To mitigate these issues and enable effective process monitoring, preliminary time series modeling using XGBoost and Support Vector Regression (SVR) will be conducted before subsequent analyses.

### Data modeling

**Fig. 4:**
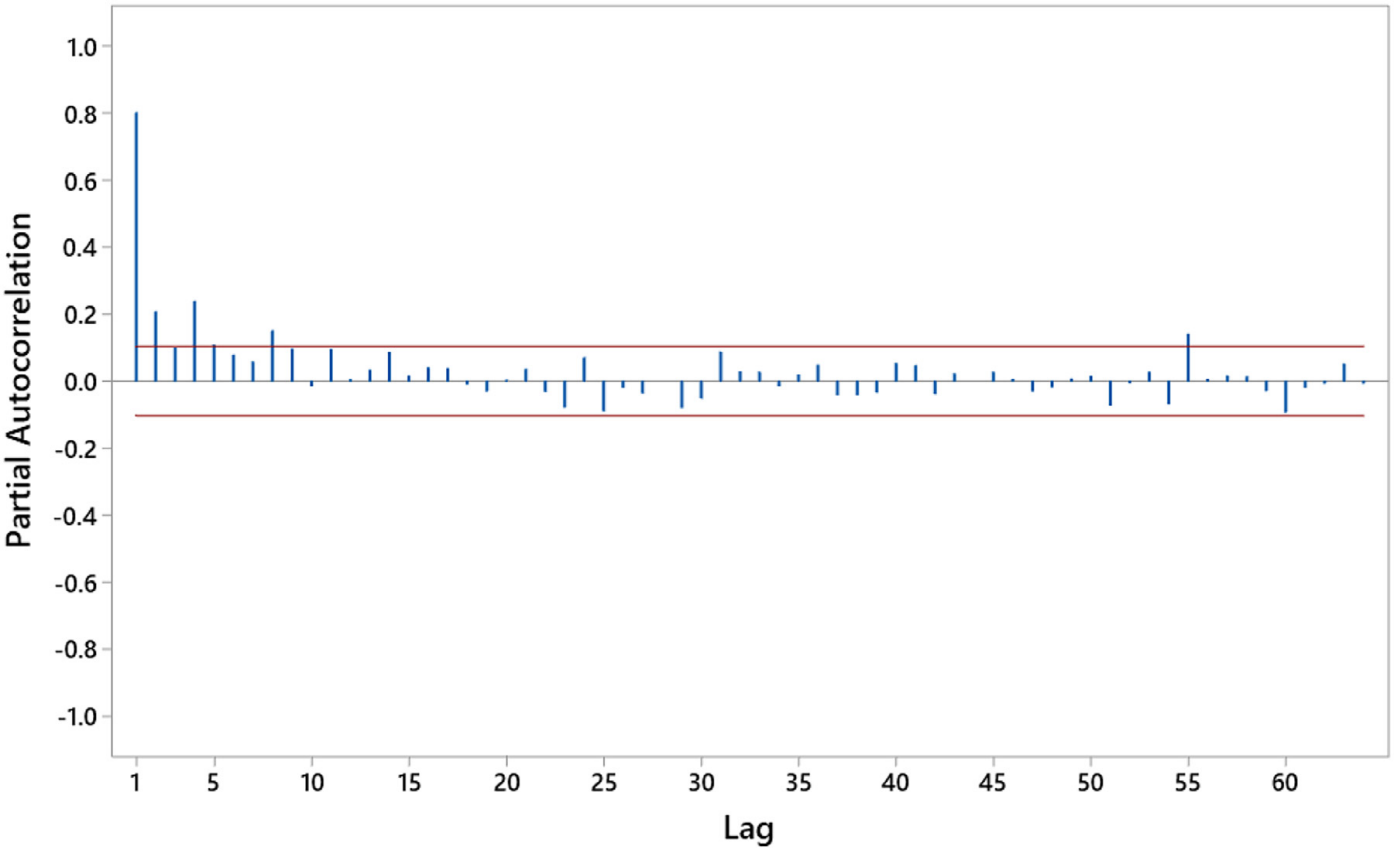
ACF Plot of air quality index data Phase I.

Modeling using Xtreme Gradient Boosting Regression and Support Vector Regression is employed to address the autocorrelation in the air quality index data based on PM 2.5 in Jakarta. The Partial Autocorrelation Function (PACF) plot is used to identify significant lags for determining the input data for both models, XGBoost and SVR. Fig. 4 shows the PACF plot of the air quality index data. Lags in PACF chosen because they displayed high partial autocorrelation values, indicating a stronger relationship with its own past values over time. This selection balances model interpretability and predictive power by capturing meaningful patterns without overfitting.

Based on Fig. 4, the PACF plot of the Phase I data with a significance level of 0.05 is significant at lags 1, 2, 4, 5, and 8. The input model for Phase I is thus $y_{\left(i-1\right)},y_{\left(i-2\right)},y_{\left(i-4\right)},y_{\left(i-5\right)},y_{\left(i-8\right)}$. Subsequently, modeling using XGBoost and SVR is conducted.

• Modeling Using Xtreme Gradient Boosting Regression

**Fig. 5:**
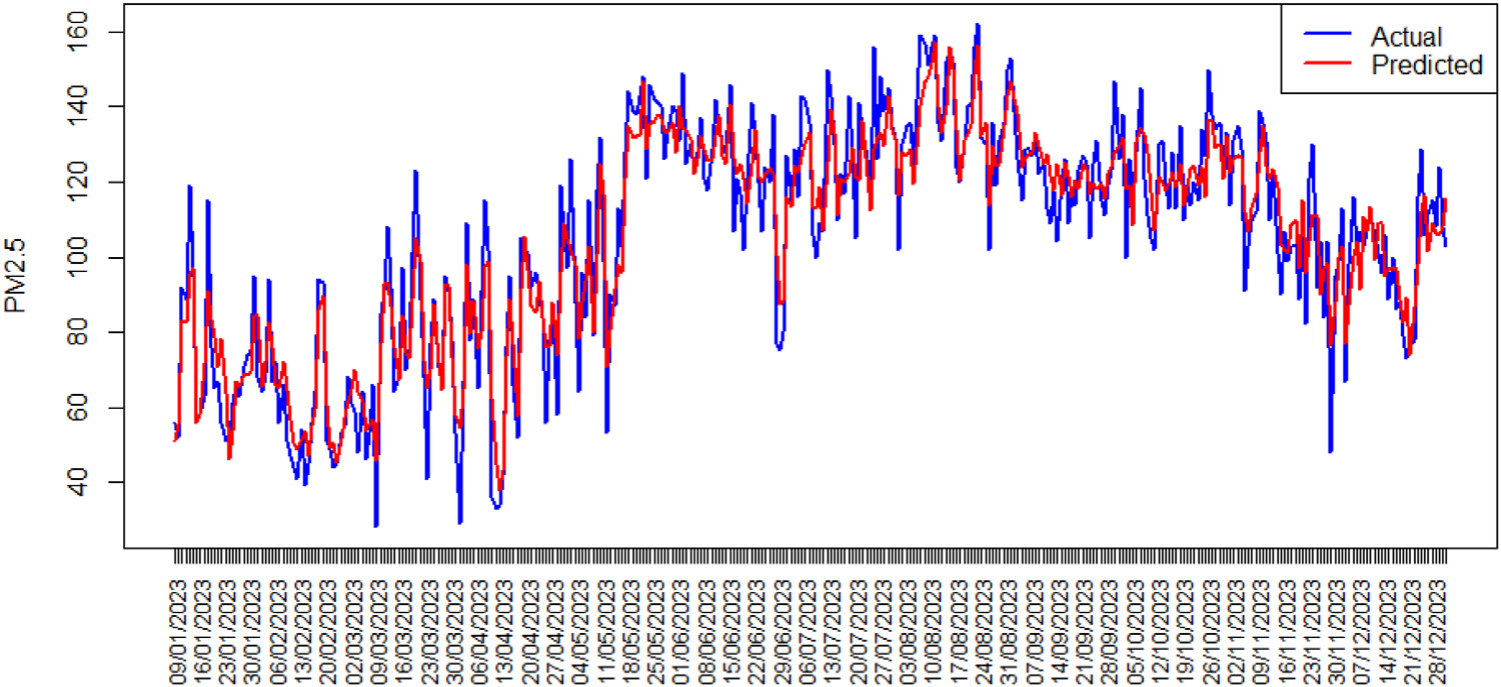
Time Series Plot Actual Data and Predicted Value XGBoost Model Phase I.

To address autocorrelation in air quality index data for each phase, an XGBoost model was employed. Following model development using observational data, predicted values (Y) were generated. A comparative analysis of predicted and observed values for phase I is visually represented in Fig. 5.

As depicted in Fig. 5, the predicted values generated by the XGBoost model for phase I exhibit a strong correlation with the corresponding actual data points. This alignment was achieved using a maximum tree depth of 3 and 50 iterations. The model's efficacy in predicting air quality index values is evident, as demonstrated by its close adherence to the observed data pattern. The low root mean squared error (RMSE) value of 9.87 with 95 % CI [9.10, 10.57] further supports the model's accuracy, indicating a minimal discrepancy between predicted and actual values.

**Fig. 6:**
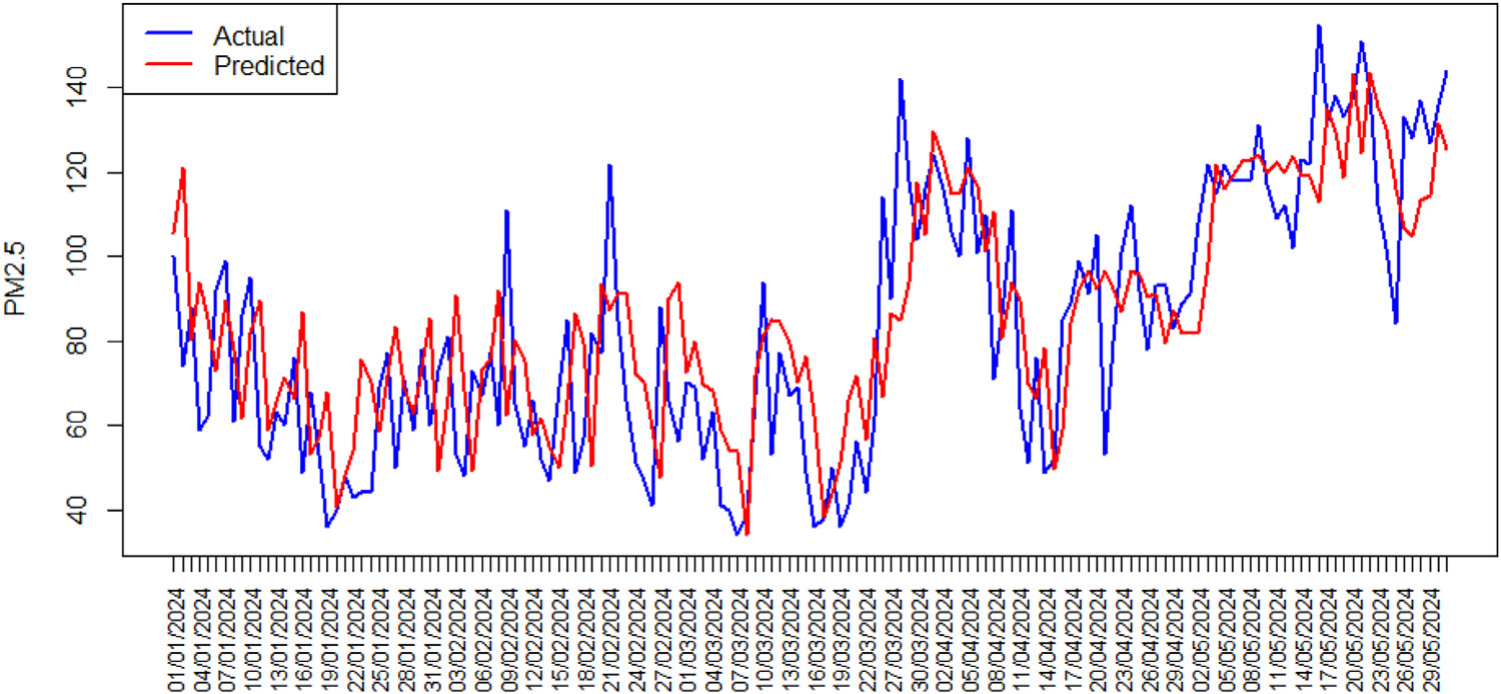
Time Series Plot Actual Data and Predicted Value XGBoost Model Phase II.

Fig. 6 shows the time series plot actual data and predicted value from XGBoost Model in phase II. Similarly, using XGBoost regression modeling on phase II data, the predicted values from the XGBoost model exhibit a pattern similar to the actual data values for phase II. Based on the obtained XGBoost model, the residual values can be effectively used to monitor the air quality index data, as the model accurately follows the actual data pattern.

Support Vector Regression (SVR) Model Performance

• Modeling Using Support Vector Regression

The air quality index data for each phase is modeled using Support Vector Regression (SVR) to address autocorrelation. After organizing the data into input variable compositions, the optimal hyperparameter values for the SVR model are determined according to the kernel type used. The kernels employed in this study are Radial Basis Function (RBF) and Polynomial kernels. For the Radial Basis Function kernel, the hyperparameters considered are *C* and σ. Specifically, the values of the hyperparameter C (cost) used are 0.01, 0.1, 1, 10, and 100, while the values of the hyperparameter σ (sigma) used are 0.01, 1, and 5. Based on these specified hyperparameter values, there are 15 combinations of hyperparameters for the Radial Basis Function kernel that will be used in a grid search to produce the best model with the smallest RMSE (Root Mean Squared Error) value. A combination of RBF Kernel Hyperparameters are presented in Table 1.

**Table 1:** Combination of RBF kernel hyperparameters.

Combination	C	*σ*
1	0.01	0.01
2	0.01	1
3	0.01	5
4	0.1	0.01
5	0.1	1
6	0.1	5
7	1	0.01
8	1	1
9	1	5
10	10	0.01
11	10	1
12	10	5
13	100	0.01
14	100	1
15	100	5

In determining the best combination of hyperparameters, all predefined hyperparameter combinations are applied to Phase I data and validation data using grid search combined with time series cross-validation. Therefore, in the time series cross-validation process, the data order is not shuffled as in cross-validation for cross-sectional data; instead, the data order remains controlled according to the time sequence to preserve information from each time period. The results of the grid search using the Radial Basis Function kernel applied to the model are presented in Table 2.

**Table 2:** RBF kernel grid search results.

C	σ	RMSE
10	0.01	17.155

In addition to using the Radial Basis Function kernel, the Polynomial kernel will also be used. For the Polynomial kernel, the hyperparameters are C, d, and r. The values for the hyperparameter C (cost) are 0.01, 0.1, 1, and 100. The values for the hyperparameter d (degree) are 1, 2, 3, 4, and 5. The values for the hyperparameter r (coefficient) are 0.001, 0.01, and 0.1. Thus, for the Polynomial kernel, there are 60 combinations of hyperparameters that will be used to obtain the optimal model through grid search. A combination of Polynomial Kernel Hyperparameters are presented in Table 3.

**Table 3:** Combination of polynomial kernel hyperparameters.

Combination	C	D	r	Combination	C	d	r
1	0.001	1	0.001	31	1	1	0.001
2	0.001	1	0.01	32	1	1	0.01
3	0.001	1	0.1	33	1	1	0.1
4	0.001	2	0.001	34	1	2	0.001
5	0.001	2	0.01	35	1	2	0.01
6	0.001	2	0.1	36	1	2	0.1
7	0.001	3	0.001	37	1	3	0.001
8	0.001	3	0.01	38	1	3	0.01
9	0.001	3	0.1	39	1	3	0.1
10	0.001	4	0.001	40	1	4	0.001
11	0.001	4	0.01	41	1	4	0.01
12	0.001	4	0.1	42	1	4	0.1
13	0.001	5	0.001	43	1	5	0.001
14	0.001	5	0.01	44	1	5	0.01
15	0.001	5	0.1	45	1	5	0.1
16	0.1	1	0.001	46	100	1	0.001
17	0.1	1	0.01	47	100	1	0.01
18	0.1	1	0.1	48	100	1	0.1
19	0.1	2	0.001	49	100	2	0.001
20	0.1	2	0.01	50	100	2	0.01
21	0.1	2	0.1	51	100	2	0.1
22	0.1	3	0.001	52	100	3	0.001
23	0.1	3	0.01	53	100	3	0.01
24	0.1	3	0.1	54	100	3	0.1
25	0.1	4	0.001	55	100	4	0.001
26	0.1	4	0.01	56	100	4	0.01
27	0.1	4	0.1	57	100	4	0.1
28	0.1	5	0.001	58	100	5	0.001
29	0.1	5	0.01	59	100	5	0.01
30	0.1	5	0.1	60	100	5	0.1

The results of the grid search combined with time series cross-validation for the Polynomial kernel applied to the model are presented in Table 4.

**Table 4:** Polynomial kernel grid search results.

C	D	r	RMSE
100	1	0.001	17.167

**Fig. 7:**
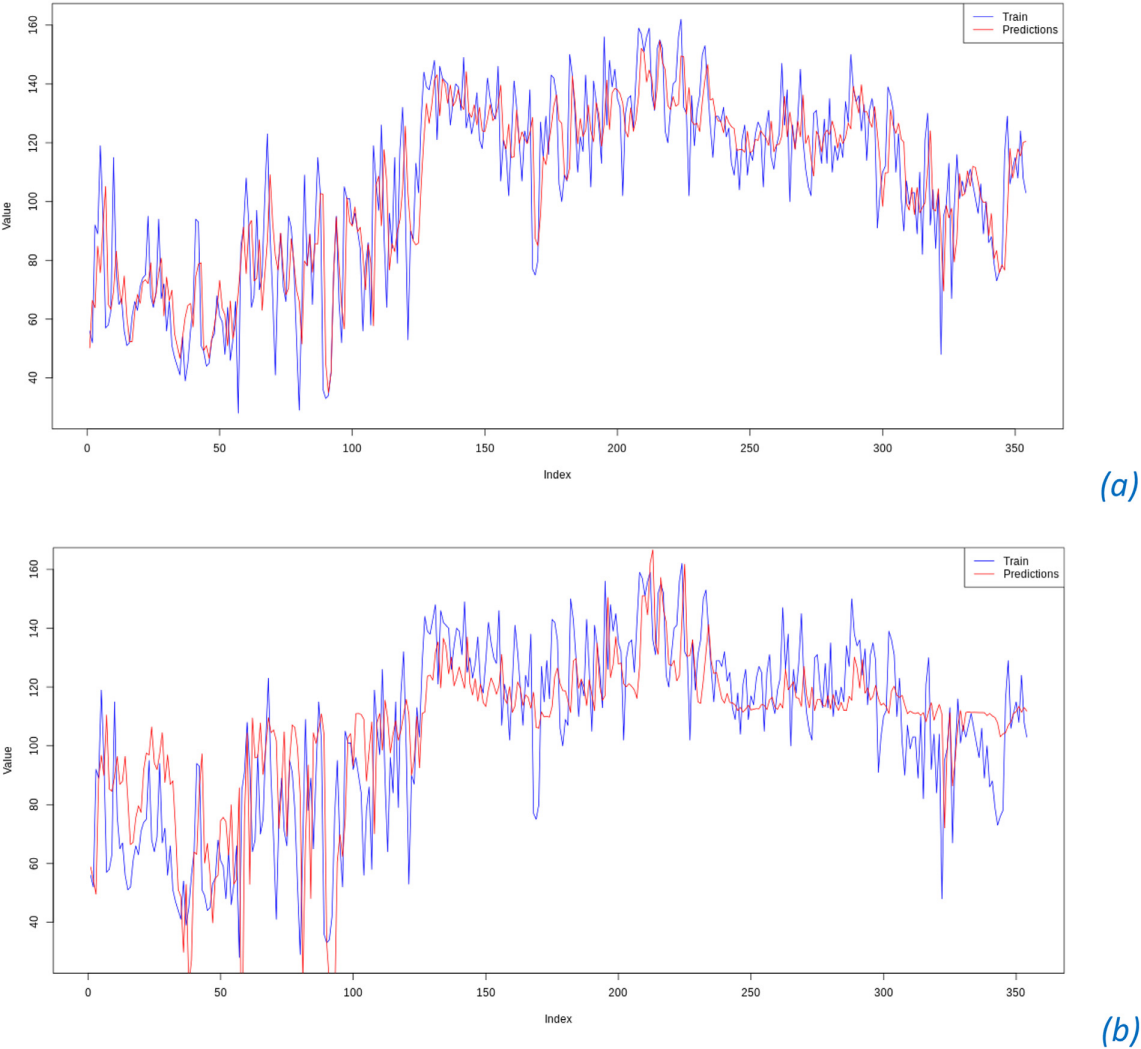
Time Series Plot Actual Data and Predicted Value SVR Model Phase I (a) RBF Kernel, (b) Polynomial Kernel.

After obtaining the best hyperparameter combinations for the Radial Basis Function and Polynomial kernels, which are applied to the model, the plots of actual vs. predicted data for Phase I are shown in Fig. 7.

Fig. 7(a) shows that the data pattern for the SVR model using the RBF kernel with the optimal hyperparameter combination of C = 10 and σ = 0.01 closely matches the actual data values for Phase I. In contrast, Fig. 7(b) shows that the data pattern for the SVR model using the polynomial kernel with the optimal hyperparameter combination of C = 100, *d* = 1, and *r* = 0.001 differs significantly from the actual data values for Phase I. These results are also consistent with the RMSE values produced by each scheme. The SVR model using the RBF kernel has a lower RMSE value of 17.155 compared to the RMSE value of 17.167 produced by the SVR model using the Polynomial kernel.

**Fig. 8:**
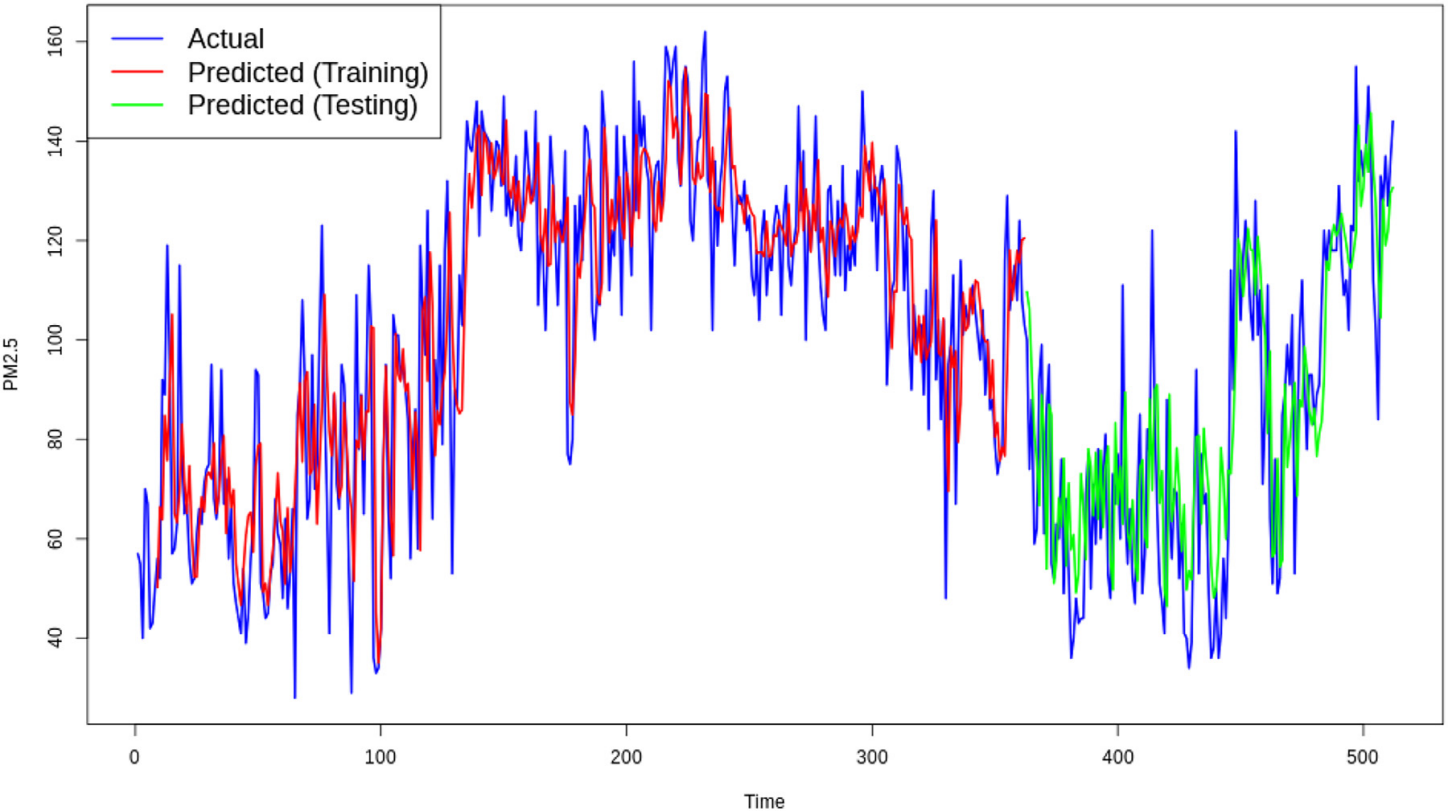
Time Series Plot Actual Data and Predicted Value SVR Model Phase II.

Fig. 8 shows the time series plot actual data and predicted value from SVR Model in phase II. Similarly, using SVR modeling on phase II data, the predicted values from the SVR model exhibit a pattern similar to the actual data values for phase II. Based on these results, the residuals of the SVR model with the RBF kernel and the optimal hyperparameter combination of C = 10 and σ = 0.01 will be used for monitoring air quality index data.

• Comparison of XGBoost Regression and Support Vector Regression

Based on the Table 5, the RMSE (Root Mean Squared Error) values for XGBoost Regression and Support Vector Regression were compared across two phases (Training and Testing). XGBoost Regression showed a lower RMSE during Training (9.87) compared to Support Vector Regression (17.16), indicating better performance initially. However, during Testing, XGBoost Regression's RMSE increased significantly to 20.38, while Support Vector Regression maintained a more consistent RMSE of 18.85. This suggests that although XGBoost Regression performed better during Training, it exhibited overfitting with a substantial drop in performance during Testing. In contrast, Support Vector Regression demonstrated more stable performance with a lower RMSE in the Testing phase, indicating better generalization to unseen data. Therefore, Support Vector Regression appears to be the more reliable model overall.

**Table 5:** Comparison of the two models.

Model	Fase	RMSE	Lower 95 % CI	Upper 95 % CI
XGBoost Regression	Fase 1 (Training)	9.87	9.10	10.57
	Fase 2 (Testing)	20.38	17.99	22.47
Support Vector Regression	Fase 1 (Training)	17.16	15.69	18.69
	Fase 2 (Testing)	18.85	16.95	20.78

The testing phase, or Phase II, is conducted to validate the performance of the developed models and ensure their applicability for prediction purposes. During this phase, the models are evaluated using unseen data to assess their ability to generalize beyond the training dataset. To numerically measure the reliability of the models, the Root Mean Square Error (RMSE) is utilized as a key performance metric. RMSE quantifies the deviation between the observed and predicted values, providing a clear indication of the model's accuracy. A lower RMSE value reflects better model performance and higher predictive reliability, making it an essential criterion for evaluating the effectiveness of the XGBoost and SVR-based residual analysis in monitoring the Air Quality Index.

• Monitoring Residual Using Individual Control Chart
• Monitoring Residual XGBoost

*Phase-I*

**Fig. 9:**
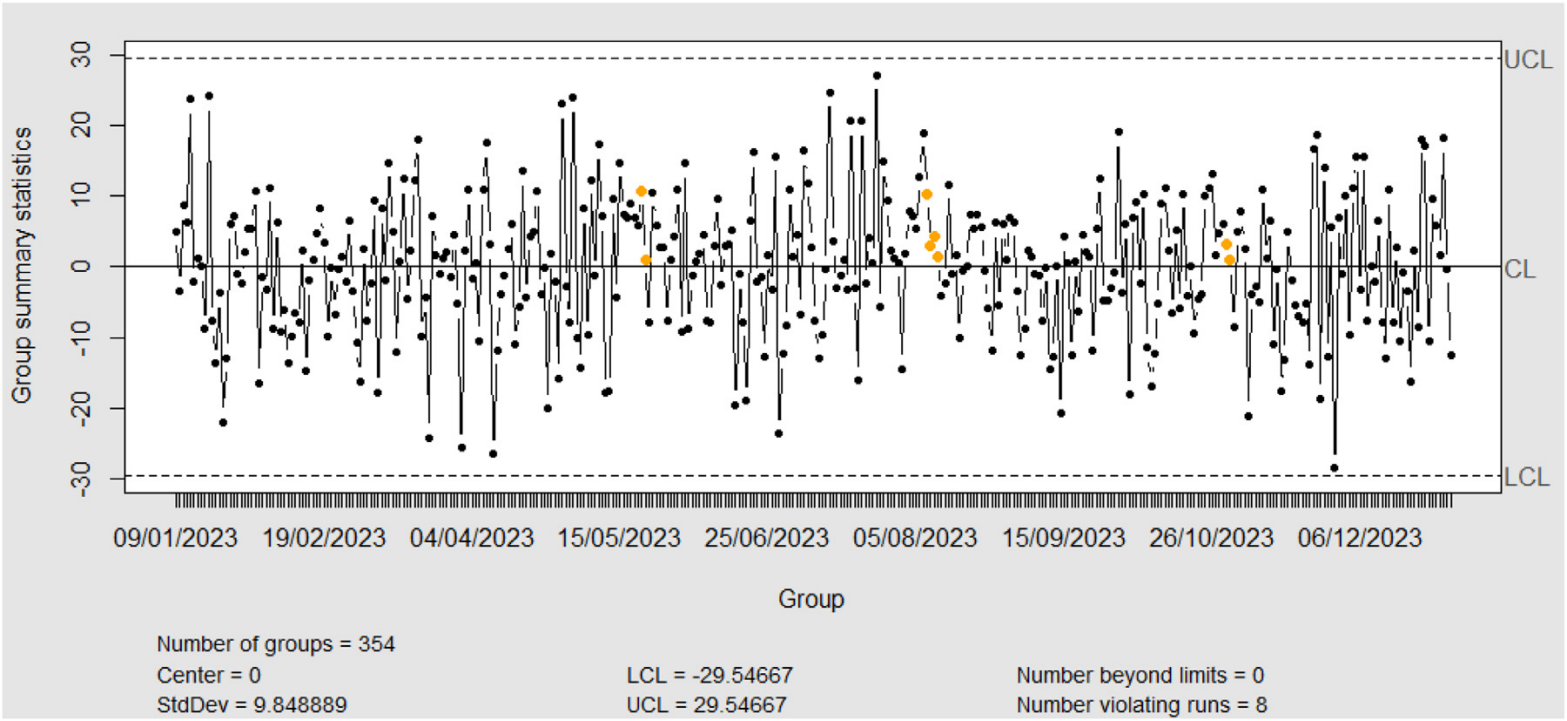
Individual Chart Phase-I from XGBoost.

**Fig. 10:**
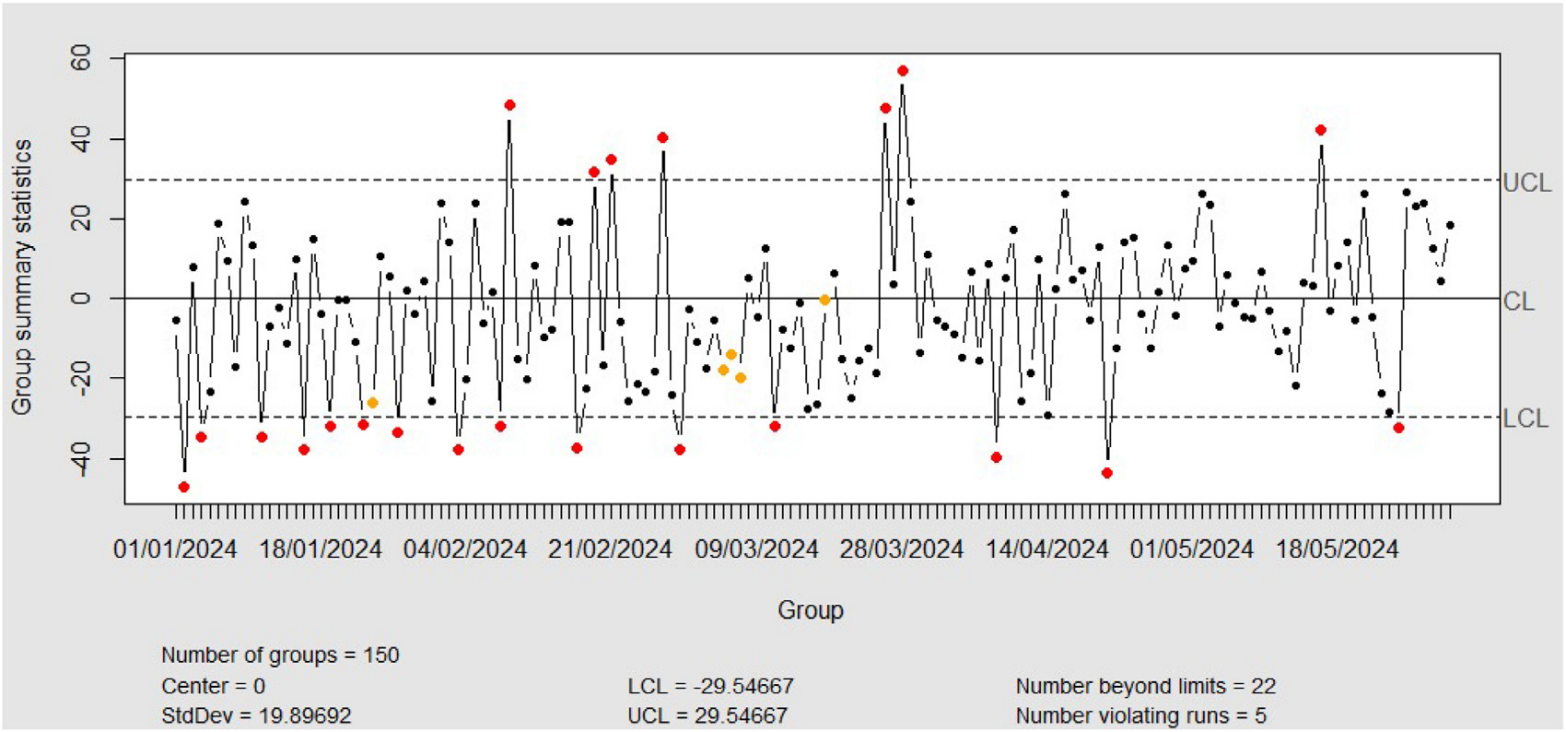
Individual Chart Phase-II from XGBoost.

Fig. 9, Fig. 10 depict the individual chart of residual phase I dan II data from XGBoost model. With no out-of-control points on the Individual chart in phase I, it indicates that there are no major shifts in the process, including in the Particulate Air Quality (PMQI) 2.5 indicator in Jakarta city. This absence of major shifts indicates that the PM 2.5 fine particle pollution level was within acceptable limits during phase I. Since the Individual chart shows good stability and control, the data and parameters from phase I can be directly used to monitor phase II.

*Phase-II*

In phase II, the Individual chart analysis showed that there was one point that was outside the control limits (OOC), indicating a considerable shift in the process. The detection of this OOC indicates unanticipated variability and needs to be investigated immediately. Some of the factors that can cause this shift include high air temperatures, weather conditions such as rain or strong winds, and increased industrial activity or vehicle traffic. Other factors such as forest fires, construction, or changes in local environmental policies also need to be considered. Further investigation is required to identify the exact cause of the detected shifts. Additional data collection on environmental conditions, human activities and weather during the period will help in a more in-depth analysis. With a better understanding of the causes of these shifts, appropriate corrective actions can be taken to bring the process back into statistical control, ensuring air quality monitoring remains effective and reliable in the city.

• Monitoring Residual SVR

*Phase-I*

**Fig. 11:**
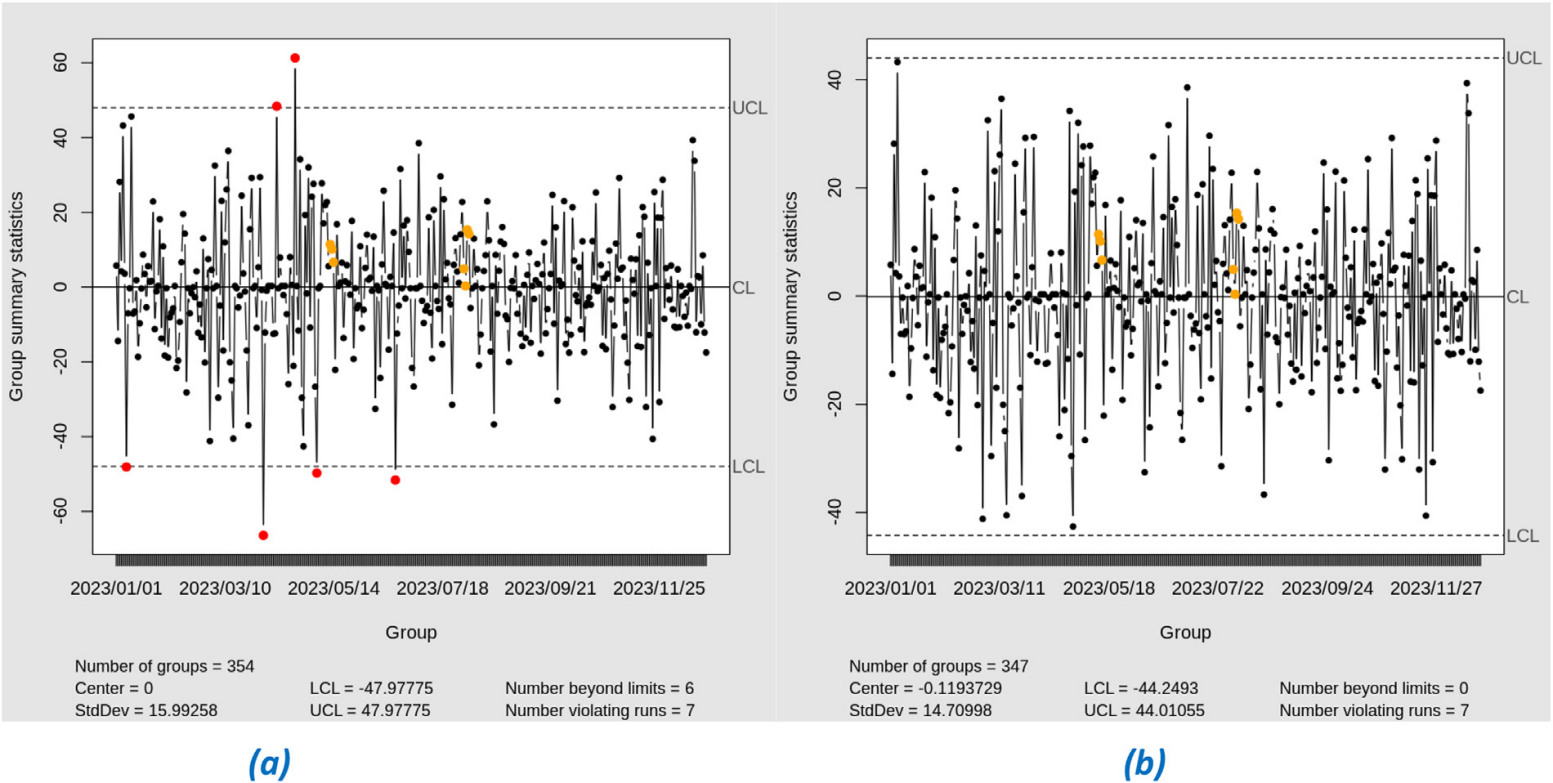
Individual Chart Phase-I from SVR (a) Out of Control (b) In Control.

**Fig. 12:**
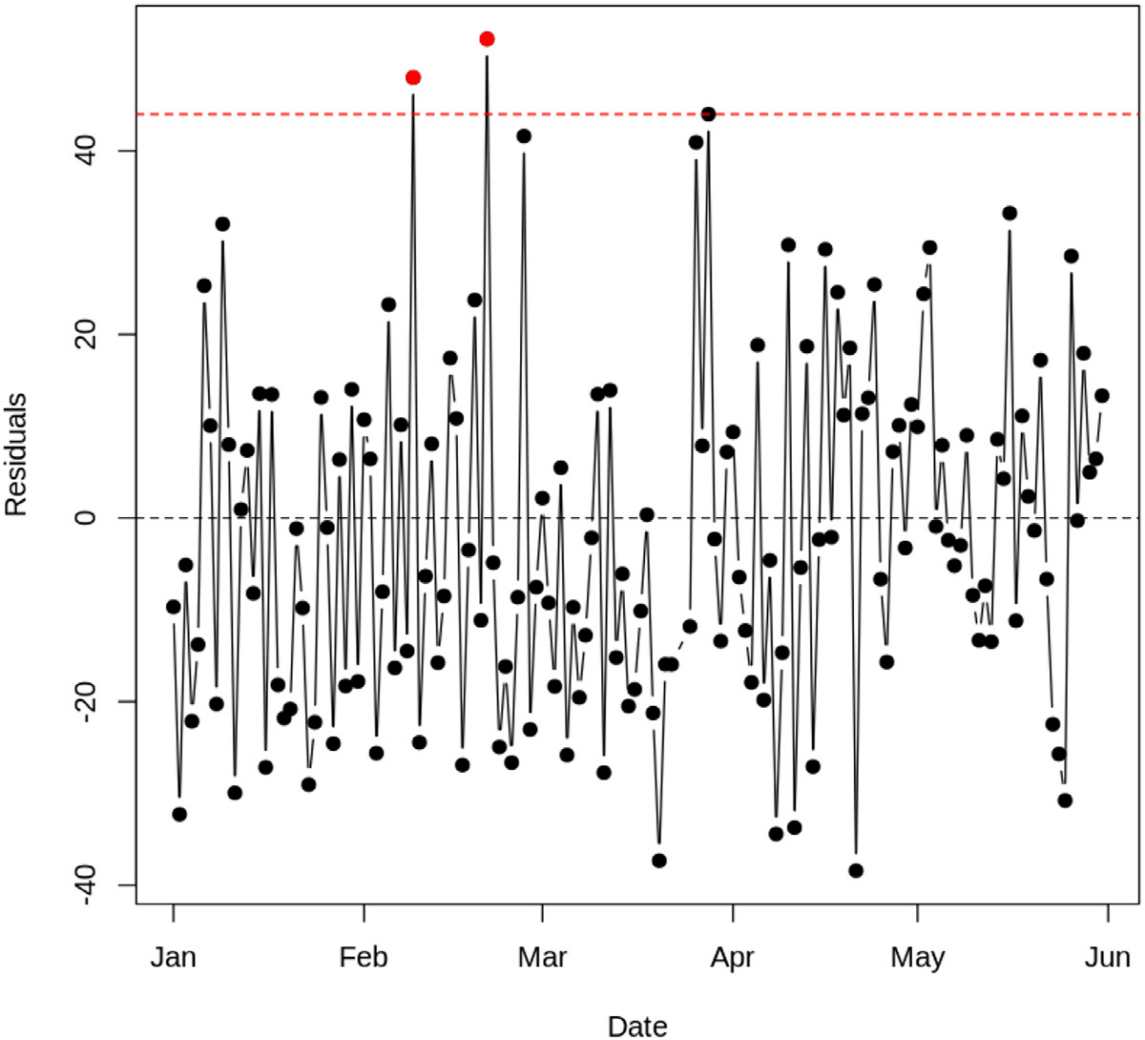
Individual Chart Phase-II from SVR.

Fig. 11, Fig. 12 depict the individual chart of residual phase I dan II data from SVR model. In phase I, modeling using Support Vector Regression (SVR) monitored with Individual charts resulted in six points that were out-of-control (OOC). These detections indicated a small shift in the process that needed to be addressed to ensure stability. Through seven treatments, all OOC points were successfully controlled, indicating that the shifts were small ones that could be detected and addressed quickly by the Individual chart. The Individual chart's sensitivity in detecting small variability helps in keeping the process in statistical control.

After ensuring there were no more OOC points in phase I, these adjusted upper and lower limits were then used for monitoring in phase II. The use of predefined control limits in phase I helps in maintaining consistency and reliability of monitoring in subsequent phases. Thus, the process can be continuously monitored with an appropriate level of sensitivity, ensuring that any small shifts that occur during phase II can be immediately detected and addressed, maintaining the overall quality and stability of the process.

*Phase-II*

In phase II, the monitoring results using the Individual chart showed that there were 2 points that were outside the control limits (out-of-control, OOC), indicating that there was a significant shift in the process during the period from January 1, 2024, to May 2024. This result suggests that the process conditions in this phase experienced considerable variability that was identified by the Individual chart. The presence of OOC points during this period indicates that the parameters and conditions set from the previous phase were not sufficient to maintain process control and reliability under the new conditions. This highlights the need for further investigation and potential adjustments to the Support Vector Regression (SVR) model to ensure it can provide consistent and reliable predictions. Therefore, while the Individual chart remains an effective tool for monitoring, the detection of these OOC points underscores the importance of continuous evaluation and adaptation to maintain process quality and stability over time.

• Monitoring Residual Using EWMA Control Chart
• Monitoring Residual XGBoost

*Phase-I*

**Fig. 13:**
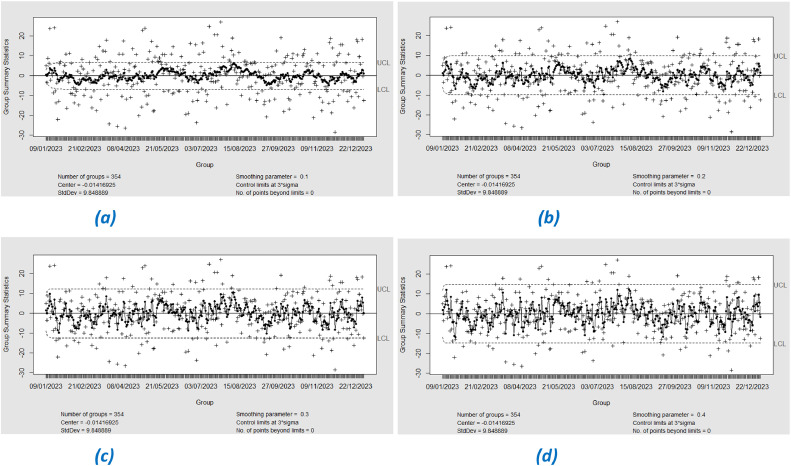
EWMA Chart Phase-I with lambda (a) 0.1, (b) 0.2, (c) 0.3, (d) 0.4.

In this study, we applied Exponential Weighted Moving Average (EWMA) control charts to monitor the residuals of the XGBoost model during the training stage. EWMA was used with varying lambda (λ) values from 0.1 to 0.4 to observe the sensitivity of the chart to changes in the residual data. The EWMA control chart with lambda (λ) 0.1 to 0.4 shows that all residual points are within the control limits (See Fig. 13). This indicates that the model does not suffer from significant variability and the prediction performance is stable. The small lambda value makes the chart more sensitive to small changes, but no indication of out-of-control (OOC) points was detected. So it can be said that in IPM 2.5 there is no significant change during the period from January 1, 2023 to December 31, 2023.

*Phase-II*

**Fig. 14:**
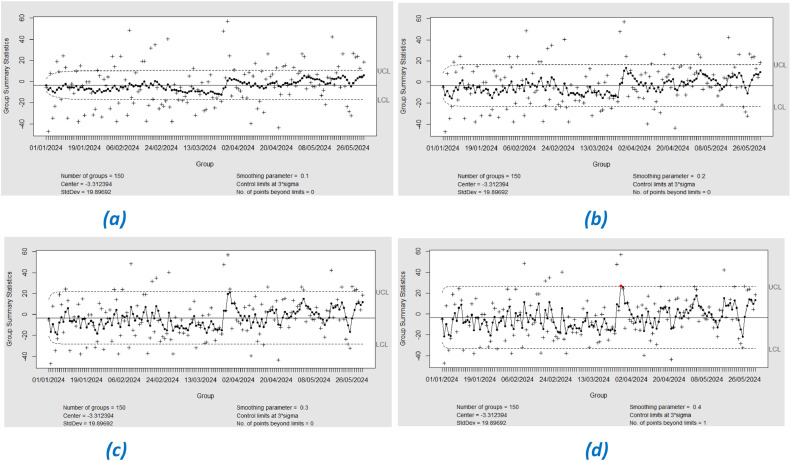
Testing EWMA Chart with lambda (a) 0.1, (b) 0.2, (c) 0.3, (d) 0.4.

In the testing stage, the application of the EWMA control chart with varying lambda values showed a shift in the process that was not detected in the training stage. In particular, with λ = 0.4, one residual point that was outside the control limits (out-of-control, OOC) was detected (See Fig. 14). This detection indicates a moderate shift in the test data. This needs to be investigated further, and the cause should be determined whether it is due to the dry season or the increase in temperature. Furthermore, it is important to note that Southeast Asia, including Jakarta, experienced a significant heatwave from March to April. This heatwave could have contributed to the observed shift in the data. The extreme temperatures during this period may have affected air quality and increased the levels of particulate matter (PM 2.5), leading to the out-of-control point detected in the EWMA control chart. This potential impact of the heatwave underscores the need for a thorough investigation into the underlying causes of the process shift. Table 6 shows the results of the EWMA chart Phase I and Phase II.

**Table 6:** Results of the EWMA chart based on XGBoost residual phase I and phase II.

Lambda	Number of OOC Phase I	Number of OOC Phase II
0.1	0	0
0.2	0	0
0.3	0	0
0.4	0	1

From the monitoring results using Exponential Weighted Moving Average (EWMA) control charts with varying lambda (λ) values from 0.1 to 0.4 in phase I, for both Support Vector Regression (SVR) and XGBoost models, it can be concluded that there is a shift in the process detected at λ = 0.4. This detection indicates a moderate to large shift, as it is only visible at higher lambda values, which are less sensitive to small changes but more sensitive to more significant changes.

• Monitoring Residual SVR

*Phase-I*

**Fig. 15:**
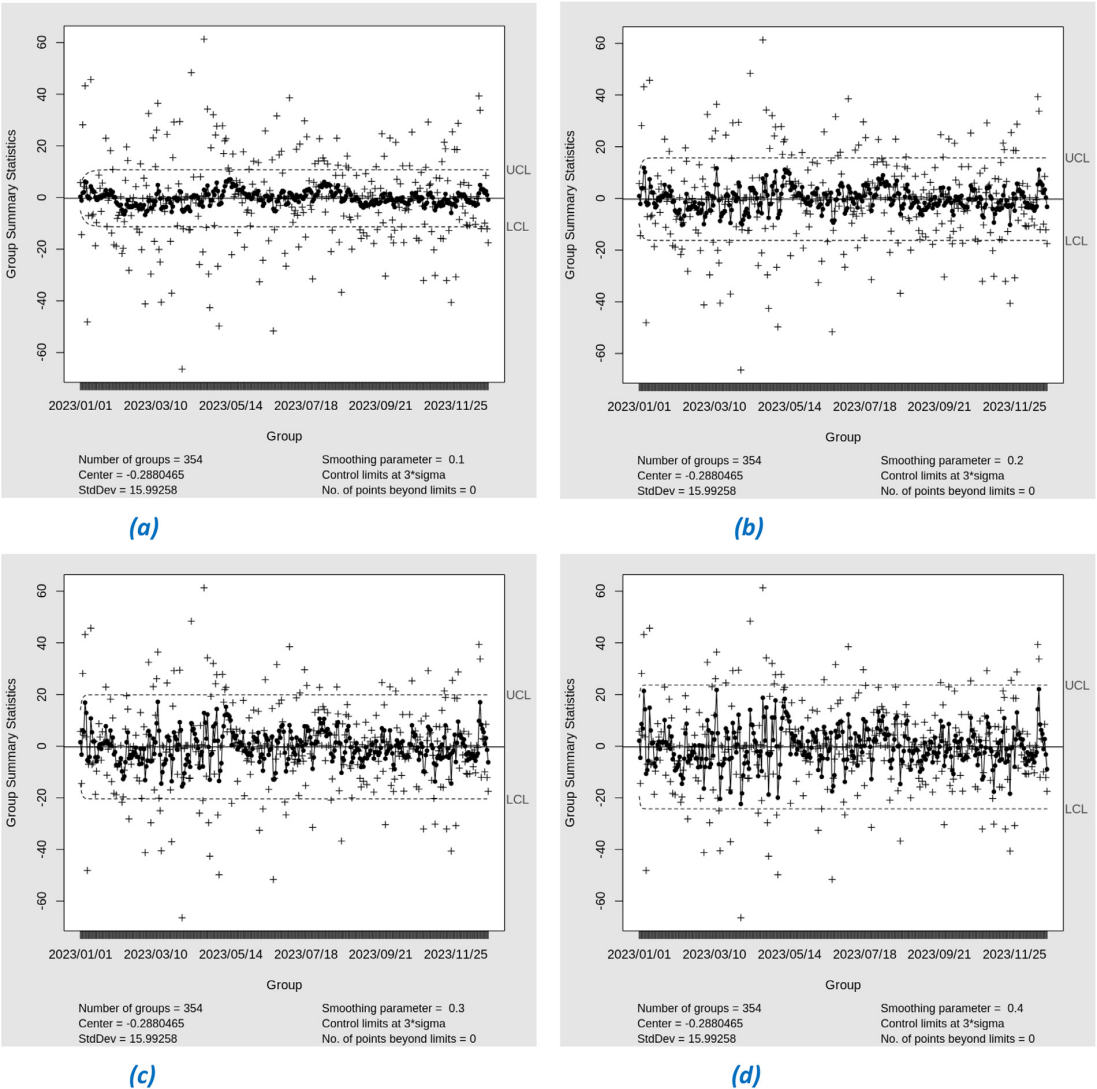
EWMA Chart Phase-I with lambda (a) 0.1, (b) 0.2, (c) 0.3, (d) 0.4.

In this study, we apply Exponential Weighted Moving Average (EWMA) control charts to monitor the residuals of the Support Vector Regression (SVR) model during the training stage. EWMA was used with varying lambda (λ) values from 0.1 to 0.4 to observe the sensitivity of the charts to changes in the residual data. The EWMA control chart with λ = 0.1 to 0.4 shows that all residual points are within the control limits (in-control) as shown in Fig. 15. This indicates that the SVR model does not experience significant variability and the prediction performance is stable during phase I. The small lambda value makes the chart more sensitive to small changes, but no indication of out-of-control (OOC) points was detected. Thus, it can be concluded that there is no significant change in IPM 2.5 during the period from January 1, 2023 to December 31, 2023. The absence of out-of-control points on the EWMA control chart during phase I indicates that the SVR model parameters and process conditions used in phase I are sufficiently stable and can be applied directly to phase II.

*Phase-II*

**Fig. 16:**
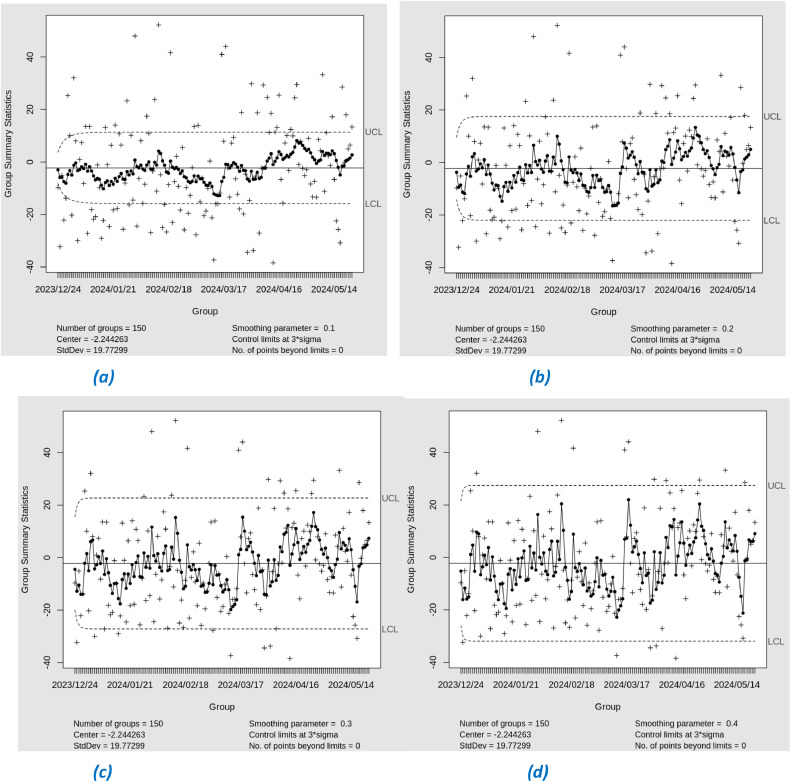
EWMA Chart Phase-II with lambda (a) 0.1, (b) 0.2, (c) 0.3, (d) 0.4.

In phase II, we applied Exponential Weighted Moving Average (EWMA) control charts to monitor the residuals of the Support Vector Regression (SVR) model. EWMA was used with varying lambda (λ) values from 0.1 to 0.4 to observe the sensitivity of the charts to changes in the residual data (See Fig. 16). The results of the analysis showed that during phase II, no residual points were out-of-control (OOC). Interestingly, even during the heatwave period that hit Southeast Asia, including Jakarta, from March to April, there were no OOC detections on the EWMA control chart. This significant heatwave has the potential to increase air temperature and affect air quality, particularly PM 2.5 particulate levels. However, the absence of OOC detection during this period may indicate flaws in the SVR model or in the EWMA parameters used, as the model should have detected significant changes in PM 2.5. Table 7 shows the results of the EWMA Phase I and Phase II.

**Table 7:** Results of the EWMA chart based on SVR residual phase I and phase II.

Lambda	Number of OOC Phase I	Number of OOC Phase II
0.1	0	0
0.2	0	0
0.3	0	0
0.4	0	0

Meanwhile, in the Support Vector Regression (SVR) model, there were no out-of-control (OOC) points in either phase II or phase II. The absence of OOC points in SVR suggests that the model may be less sensitive in detecting shifts, including moderate to large shifts. This indicates that SVR may not be responsive enough to significant changes in the residual data, which should be detected by the control chart.

SVR-EWMA showed no out-of-control (OOC) points in either phase II or phase II, indicating a potential limitation in detecting shifting. This suggests that SVR-EWMA modeling may be less sensitive in small shift in the residual data, which a control chart is intended to highlight. This could be due to SVR's tendency to fit the data more closely, sometimes resulting in overfitting and reducing its effectiveness in capturing shifts. In contrast, XGBoost follows broader data patterns, leading to a more robust control chart model. As a result, XGBoost demonstrated better performance in detecting OOC points when combined with EWMA. Although SVR achieved a lower RMSE, showing superior predictive accuracy for time series modeling, XGBoost excelled in control applications by effectively identifyng shifts in the data.

### Comparison results

After obtaining the Individual control chart and EWMA control chart based on the residuals of the XGBoost model and SVR model, the next step is to compare these control charts to determine which one is more sensitive to process shifts in each phase, as indicated by the number of out-of-control observations. Table 8 provides a comparison of the residuals from each model, each control chart, and each phase.

**Table 8:** Comparison results.

Type of residual model	Type of control chart	Phase	The number of out of control detected
XGBoost Regression	Individual Chart	Phase I	0
		Phase II	22
	EWMA Chart	Phase I	0
		Phase II	1
Support Vector Regression	Individual Chart	Phase I	6
		Phase II	0
	EWMA Chart	Phase I	0
		Phase II	0

Based on Table 8, an analysis was conducted on the performance, stability, and sensitivity of various combinations of residual models and control charts. The XGBoost Regression paired with the EWMA Chart found no out-of-control (OOC) points in Phase I and only one in Phase II, reflecting high stability and effective sensitivity to process changes. In contrast, the XGBoost Regression with the Individual Chart identified 22 OOC points in Phase II, indicating excessive sensitivity and a likelihood of false positives. The Support Vector Regression (SVR) combined with the Individual Chart detected 6 OOC points in Phase I and II in Phase II, showing moderate sensitivity but a higher rate of false alarms compared to the EWMA Chart. The SVR with the EWMA Chart detected no OOC points in either phase, which could mean a lack of sensitivity or a very stable process. Overall, the XGBoost Regression with the EWMA Chart is the most robust combination, offering a good balance of stability and sensitivity by detecting only one OOC point in Phase II and none in Phase I. This makes it the best choice for reliable process monitoring with minimal false alarms.

This study highlights the critical role of addressing autocorrelation in air quality data to enhance monitoring and management practices. By applying XGBoost and Support Vector Regression (SVR) models to PM 2.5 data from Jakarta, we demonstrated that these advanced modeling techniques effectively handle autocorrelation and improve the accuracy of air quality predictions. The SVR and XGBoost methods effectively capture the overall data patterns. Although the dataset used is short-term, it is already representative as it encompasses two seasons in Indonesia, the dry and rainy seasons. The training data includes observations from all seasons in Indonesia, a tropical country characterized by these two distinct seasons. Similarly, the testing data also covers both seasons, with the first three months representing the rainy season and the subsequent two months representing the dry season. SVR and XGBoost are well-known methods for handling short-term data. Previous research has highlighted that XGBoost utilizes parallel processing and is highly efficient in memory usage, making it ideal for processing large datasets and developing models within a short time frame. While SVR is generally computationally intensive due to its kernel operations, the use of optimized parameters effectively addresses this issue [31].

The XGBoost model, combined with the EWMA control chart, provided the best overall performance. This combination detected only one out-of-control (OOC) point in Phase II and none in Phase I, indicating high stability and appropriate sensitivity. In contrast, the SVR model showed more stable performance across both phases but with a slightly higher rate of false alarms. These findings underscore the importance of integrating machine learning models with suitable control charts for reliable air quality monitoring. The XGBoost-EWMA combination offers a balanced approach, minimizing false alarms while maintaining sensitivity to significant shifts in air quality data. The identification of OOC points provides actionable insights by highlighting significant deviations in air quality data that may require immediate intervention. Policymakers can use these findings to investigate root causes, adjust regulations, and implement targeted solutions to mitigate air quality deterioration in Jakarta.

This study will be expanded by incorporating data with a longer time span to better capture long-term patterns and trends in air quality variations across different seasons. Additionally, future research will employ multivariate analysis methods to integrate and analyze a broader set of variables that potentially influence the air quality index, such as meteorological factors, population density, industrial activities, and transportation patterns. By doing so, the research aims to provide a more comprehensive understanding of the factors affecting air quality and enhance the predictive accuracy of the models, thereby offering more robust insights for policymakers and stakeholders in managing and improving air quality in tropical urban environments. Future research should explore the application of these methods to other pollutants and regions to validate their effectiveness further. Additionally, incorporating more sophisticated machine learning techniques and hybrid models could enhance prediction accuracy and monitoring capabilities.

## Limitations

This study has several limitations that need to be considered. First, the machine learning method used, particularly XGBoost, tends to experience overfitting when applied to long time spans, which can reduce the model's generalization ability and degrade its performance when faced with new data. Second, the control charts used in the analysis, including the Individual and EWMA charts, are only able to detect small process shifts. However, they may require further inspection to ensure that the process is truly in control, especially when dealing with more significant changes in air quality data. Air quality is typically assessed using several key pollutants as indicators, including particulate matter (PM10 and PM2.5), nitrogen dioxide (NO2), sulfur dioxide (SO2), carbon monoxide (CO), ozone (O3), and volatile organic compounds (VOCs). These pollutants are critical as they directly affect human health and the environment. For instance, PM2.5 and PM10 are associated with respiratory and cardiovascular diseases, while ground-level ozone contributes to lung damage and worsens conditions like asthma. Third, The study focused exclusively on PM2.5 concentrations in Jakarta, which may limit the generalizability of the findings to other pollutants or geographical locations. Future research could be expanded to include a broader range of air quality indicators to provide a more comprehensive assessment of air quality. Additionally, monitoring efforts should be extended to other regions in Indonesia, such as Bandung and Surabaya, which face challenges from high vehicular emissions and industrial activities, as well as Kalimantan and Sumatra, where recurring forest fires significantly impact air quality. Additionally, this study did not incorporate variables such as weather conditions, wind patterns, and other environmental factors, which may influence air quality. Future research could benefit from multivariate analysis and consider incorporating these additional variables. Lastly, the study relied on a specific set of hyperparameters and model configurations, which may not be optimal for all datasets or conditions. Further investigation is needed to identify the best practices for hyperparameter tuning and model selection in various contexts.

## Ethics statements

This research did not involve research on humans or animals, and no data is involved from social media platforms.

## Declaration of competing interest

The authors declare that they have no known competing financial interests or personal relationships that could have appeared to influence the work reported in this paper.

## Data Availability

Data will be made available on request.
